# Molecular Characterization of the N-Acetylglucosamine Catabolic Genes in *Candida africana*, a Natural N-Acetylglucosamine Kinase (*HXK1*) Mutant

**DOI:** 10.1371/journal.pone.0147902

**Published:** 2016-01-25

**Authors:** Maria Rosa Felice, Megha Gulati, Letterio Giuffrè, Domenico Giosa, Luca Marco Di Bella, Giuseppe Criseo, Clarissa J. Nobile, Orazio Romeo, Fabio Scordino

**Affiliations:** 1 Department of Biological and Environmental Sciences, University of Messina, Messina, Italy; 2 Department of Molecular and Cell Biology, University of California Merced, Merced, California, United States of America; 3 Scientific Institute for Research, Hospitalization and Health Care (IRCCS)—Centro Neurolesi "Bonino-Pulejo", Messina, Italy; IRCCS Casa Sollievo della Sofferenza Hospital, ITALY

## Abstract

**Background:**

In this study we report the genetic characterization, including expression analysis, of the genes involved in the uptake (*NGT1*) and catabolism (*HXK1/NAG5*, *DAC1/NAG2*, *NAG1*) of the aminosugar N-acetylglucosamine (GlcNAc) in *Candida africana*, a pathogenic biovariant of *Candida albicans* that is naturally unable to assimilate the GlcNAc.

**Results:**

DNA sequence analysis of these genes revealed a number of characteristic nucleotide substitutions including a unique and distinctive guanine insertion that shifts the reading frame and generates a premature stop codon (TGA) 154 bp downstream of the ATG start codon of the *HXK1* gene encoding the GlcNAc-kinase, a key enzyme of the GlcNAc catabolic pathway. However, all examined genes produced transcripts even though different levels of expression were observed among the *Candida* isolates examined. In particular, we found an *HXK1*-idependent relationship of the *NGT1* gene and a considerable influence of the GlcNAc-kinase functionality on the transcription of the *DAC1* and *NAG1* genes. Additional phenotypic analysis revealed that *C*. *africana* isolates are hyperfilamentous in the first 24-48h of growth on filament-inducing media and revert to the yeast morphological form after 72h of incubation on these media.

**Conclusions:**

Our results show that *C*. *africana* is a natural *HXK1* mutant, displaying a number of phenotypic characteristics distinct from typical *C*. *albicans* isolates.

## Introduction

*Candida africana* is a pathogenic fungus reported to cause genital infections in humans [1, 2]. It was first isolated in 1995 in Africa and subsequently proposed as a new *Candida* species closely related to the well-known fungal pathogen *Candida albicans* [3]. However, although *C*. *africana* exhibits a number of phenotypic characteristics clearly distinct from those observed in typical *C*. *albicans* isolates [3, 4], several molecular studies have demonstrated that these two fungi are, genetically, too closely related to be considered two distinct species [5, 6, 7, 8, 9]. Therefore, *C*. *africana* is currently considered to be a biovariant of *C*. *albicans* rather than a new species [1, 10].

Phenotypically, like *C*. *albicans*, *C*. *africana* produces characteristic germ-tubes in the presence of serum, however, unlike *C*. *albicans*, *C*. *africana* is unable to form chlamydospores and is unable to assimilate certain organic compounds including the two aminosugars N-acetylglucosamine and glucosamine [3].

N-acetylglucosamine (GlcNAc) is a monosaccharide essential for various cellular processes in both eukaryotic and prokaryotic organisms [11] and it is metabolized by 100% of typical *C*. *albicans* isolates [12]. In addition to utilizing GlcNAc as a carbon and energy source, in *C*. *albicans* GlcNAc also plays an important role in cell signaling by inducing white-opaque switching, the conversion between two heritable phenotypic states [13] and by modulating the yeast-to-hyphal transition [14] both of which are associated with virulence and pathogenicity in this fungus [15].

A previous proteomics study of plasma membrane proteins in *C*. *albicans* identified a unique and specific transporter (called *NGT1*) that allows for the uptake of GlcNAc in this species [16]. Once inside the cell, GlcNAc is phosphorylated, deacetylated and deaminated by three enzymes, encoded by the genes *HXK1*, *DAC1* and *NAG1*, respectively. These enzymes ultimately convert GlcNAc into fructose-6-phosphate, which is used as an energy source by the cell [11].

*C*. *africana* is a natural GlcNAc-mutant, unable to use this monosaccharide for growth [3]. Furthermore, this fungus also shows a remarkably low level of filamentation [17], poor adhesion to human cells [18] and decreased virulence using *Galleria mellonella* as an infection model [17]. Since these properties are all linked to the ability to undergo morphological transitions, which in certain conditions is, in part, dependent on extracellular GlcNAc, we decided to study the sequence and expression levels of the genes involved in the GlcNAc catabolic pathway (also called NAG regulon) [14], including the *NGT1* gene [16], in order to understand GlcNAc utilization in *C*. *africana*.

## Materials and Methods

### Fungal strains

Two *C*. *africana* strains: UPV/EHU 97135 from Bilbao, Spain [19] and CBS11016 from Centraalbureau voor Schimmelcultures, isolated in Vibo Valentia, Italy [8] were examined in this study. The identity of the strains was confirmed by partial amplification and detection of the *HWP1* gene according to previous studies [20]. The *C*. *albicans* ATCC10231 strain was used as a reference strain in all experiments.

Ten Chinese *C*. *africana* strains (2313, 2286, 8513, 8840, 8866, 8350, 8367, E373, 5006, 5344 [21]) and one from Germany (A1622 [3]) were also included and used to confirm the observed genetic polymorphisms.

### Phenotypic tests

**Fig 1:**
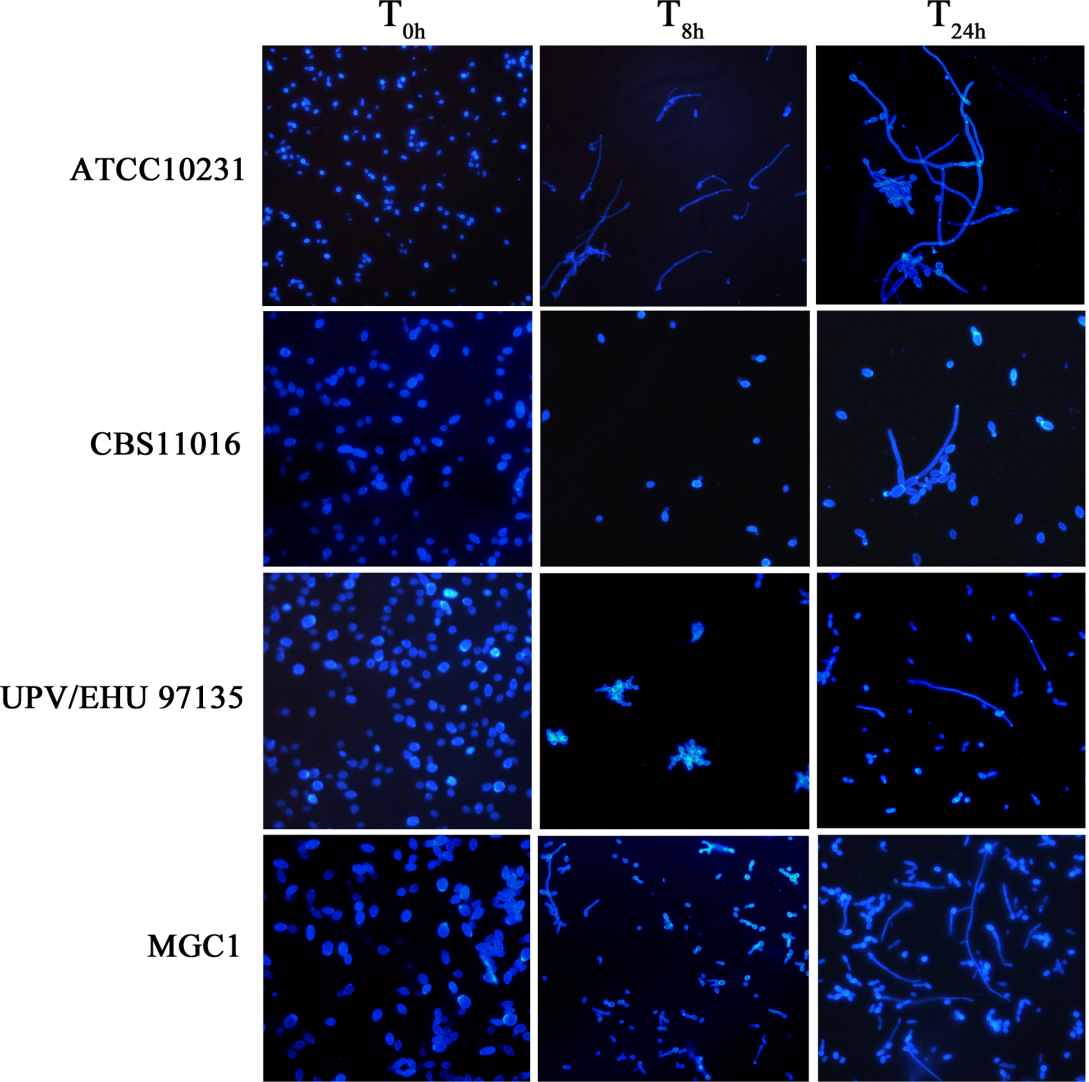
Morphologies of *C*. *albicans* and *C*. *africana* in YNB medium plus GlcNAc (5 mM final concentration). Images were taken at different intervals of time (T_0h_, T_8h_ and T_24h_). Results from different concentrations of GlcNAc (ranging from 0.195–100 mM) showed similar levels of filamentation (not shown).

To determine the extent to which *C*. *africana* is unable to grow in the presence of GlcNAc, all isolates were initially grown in liquid yeast nitrogen base (YNB) medium (Difco, Milan, Italy) containing 2% of galactose and then incubated at 30°C in the presence of different concentrations of GlcNAc (ranging from 0.195–100 mM). Assimilation tests were carried out as outlined in Kurtzman and Fell [22] using 5 ml of liquid YNB containing GlcNAc (first tube 100 mM) serially diluted to 0.195 mM. The test tubes were inoculated with 0.1 ml of a standardized fungal suspension (~10^7^cells/ml) prepared spectrophotometrically, and incubated at 30°C for up to 28 days. To examine hyphal induction in such conditions, yeast cultures were microscopically inspected at hourly intervals from 2-8h with a final reading at 24h. Fig 1 shows results of filamentation at a 5 mM final concentration of GlcNAc; however we note that all concentrations of GlcNAc tested produced similar levels of filamentation. Filamentation was further assayed using YEPD agar plus 10% fetal bovine serum (Sigma-aldrich, Milan, Italy) and Spider medium (nutrient broth 20 g/L, mannitol 20 g/L, K_2_HPO_4_ 4 g/L, Bacto agar 27 g/L; pH 7.2) [23]. On these plates, 3 μl of standardized fungal suspension that contained 10^4^, 10^3^, 10^2^ and 10 cells/μl, respectively, were spotted and plates were incubated, in duplicate, at 30 and 37°C for 7 days. Standard YEPD agar plates without serum were inoculated and used as a control.

100 μl of the standardized fungal suspensions (~10^7^ cells/ml) were also used to inoculate 5 ml of YNB supplemented with 50 mM of galactose and 10 mM of GlcNAc in order to assess if our *C*. *africana* strains could be considered as *NAG1* and/or *DAC1* mutants according to previous studies [24].

### Molecular tests

#### PCR primer design, amplification and sequencing of the *NGT1* gene and NAG regulon

All PCR primers used to amplify and sequence the entire open reading frame (ORF) of the *NGT1*, *HXK1*, *DAC1* and *NAG1* genes are listed in Table 1.

**Table 1:** Primers used for amplification, sequencing and expression analysis.

Primer name	Sequence (5'→3')	ORFs^a^	Amplicon size	Reference
NGT1-F	TACGGCATTGGAACAACCC	19.5392	1804 bp	This study
NGT1-R	CCAACAGGATAGATTAGCCAACC	19.5392	--	--
NGT1-2F^b^	ATGATTTGTGGATCAGTATTGGC	19.5392	968 bp	This study
NGT1-2R^c^	CGTCTTTCCAGTAAGGGTGAGT	19.5392	942 bp	This study
NAG1-F	GCATTCGGTTGTTCTGGGAG	19.2156	1098 bp	This study
NAG1-R	CCTTCAACTCTTATCTGTCGCC	19.2156	--	--
HXK1-Fa	CGCCAGTTGAATAAGATGGC	19.2154	1178 bp	This study
HXK1-Ra	GGCGACAGATAAGAGTTGAAGG	19.2154	--	--
HXK1-Fb^d^	CGGTGTATTCTGCTGGGTG	19.2154	898 bp	This study
HXK1-Rb^e^	CACCCAGCAGAATACACCG	19.2154	896 bp	This study
DAC1-Fa	CTCCCAGAACAACCGAATGC	19.2157	1459 bp	This study
DAC1-Ra	CGACGAATGGGCAACCTC	19.2157	--	--
DAC1-Fb^f^	TGCCACCCAGTCGAAACG	19.2157	930 bp	This study
NGT1-RT-R^g^	TGGACCGATATAAGCACCATC	19.5392	491 bp	This study
NAG1-RT-F	GCATTGAGTGTTGGTATTTCCACC	19.2156	195 bp	This study
NAG1-RT-R	ACTTTAATCCAGCGGCAGC	19.2156	--	--
HXK1-RT-F	CAAATTGTTGGGCAGTGACG	19.2154	197 bp	This study
HXK1-RT-R	TGATAGGCAGCACCTATGGC	19.2154	--	--
DAC1-RT-F	CGTAACCTAGTCAAGTGGTCGC	19.2157	198 bp	This study
DAC1-RT-R	TATCTGACGACTGGACTTCACG	19.2157	--	--
ACT1-RT-F	TCCAGAAGCTTTGTTCAGACCAGC	19.5007	170 bp	[24]
ACT1-RT-R	TGCATACGTTCAGCAATACCTGGG	19.5007	--	--

^a^Open reading frames (ORFs) were downloaded from www.candidagenome.org and used for primer design

^b^This primer was used in combination with the reverse primer NGT1-R

^c^This primer was used in combination with the forward primer NGT1-F

^d^This primer was used in combination with the reverse primer HXK1-Ra

^e^This primer was used in combination with the forward primer HXK1-Fa

^f^This primer was used in combination with the reverse primer DAC1-Ra

^g^This primer was used in combination with the forward primer NGT1-2F.

Primers used for expression analysis are labeled with "RT".

Primers were designed with the Vector NTI program (version 10.3.0; Invitrogen, Italy) using the sequenced *C*. *albicans* genome (version A22-s04-m01-r03; www.candidagenome.org). Validation of primer specificity was performed using the web-based primer-BLAST tool (www.ncbi.nlm.nih.gov/tools/primer-blast/index.cgi).

For each gene, its ORF (Table 1) plus 1 kb of upstream and downstream sequence, was downloaded in FASTA format from the *Candida* genome database (www.candidagenome.org). A primer pair spanning ~200 bp or less upstream and downstream of each ORF was designed to amplify and sequence the entire coding region of each gene and additional internal sequencing primers were designed in cases where PCR primers did not suffice to cover the whole gene region in both directions (Table 1).

Total genomic DNA was extracted using a high-speed cell disruption method followed by phenol-chloroform-isoamyl alcohol extraction and ethanol precipitation as described in Müller et al. [25].

In vitro amplifications were carried out in 50 μl volumes using DreamTaq^TM^ PCR master mix (Fermentas, Milan, Italy) plus 1 μl of genomic DNA template and 0.5 μM of each primer pair (Table 1). All PCRs were performed in a MyCycler thermal cycler (Bio-Rad, Milan, Italy) with initial denaturation step at 95°C for 5 min, followed by 35 cycles of denaturation at 94°C for 45 s, annealing at 52°C for 40 s, extension at 72°C for 90 s, and a final extension step of 10 min at 72°C.

A 3 μl aliquot of each PCR reaction was subjected to agarose gel electrophoresis to confirm the presence of amplified products, expected size and the absence of nonspecific fragments. The remaining amount of each amplicon was purified using the QIAquik PCR purification kit (Qiagen, Milan, Italy) and both strands were sequenced by MWG-Eurofins, Ebersberg, Germany (www.eurofinsgenomics.eu).

Electropherograms were inspected for errors and heterozygous mutations using FinchTV 1.4 software (www.geospiza.com) and sequence data were assembled using ContigExpress as implemented in Vector NTI.

Each DNA sequence was aligned using MEGA5 software [26] and compared with the corresponding wild-type ORF sequence (Table 1). Each nucleotide substitution leading to an amino acid change was inspected visually and recorded. Silent mutations were not considered.

All nucleotide sequences obtained in this study have been deposited in the GenBank database under the following accession numbers: KP052779, KP052780, KP165328, KP165330, KP165331, KP193952, KP193954, KP193955, KP193956, KP193958 and KP193959.

#### RNA extraction and RT-PCR assays

For gene expression studies, all isolates were grown in YNB with 2% galactose for 48h at 30°C. After incubation, cells were centrifuged at 1,455 g for 15 min, washed twice with sterile water and resuspended in YNB containing 5 mM GlcNAc. Yeast isolates were incubated at 30°C and RNA extraction was performed at 2.5, 8 and 24 hours. Total RNA was also obtained from fungal cultures grown in 2% galactose without GlcNAc induction.

RNA extraction from *Candida* spp. cultures was obtained by EuroGold Trifast reagent (EuroClone, Milan, Italy) following the manufacturer’s instructions with minor modifications. In brief, yeast cells were harvested by centrifugation at 1,455 g for 20 min, the pellet was resuspended in 1 ml of reagent and cells were lysed by mixing with an equal volume of acidified RNase-free glass beads (Sigma-Aldrich, Milan, Italy). One microgram of total RNA was subjected to DNaseI digestion following the manufacturer’s instructions (Sigma-Aldrich, Milan, Italy) and was reversely-transcribed using BioScipt M-MLV-Retro Transcriptase (Bioline, London, UK) with oligodT primer (EuroClone, Milan, Italy) at 42°C for 60 min followed by a denaturation step of 15 min at 70°C. Single strand cDNA was used in the PCR reaction to amplify *NGT1*, *HXK1*, *DAC1*, *NAG1* and *ACT1* (actin; housekeeping control) genes using BioTaq DNA polymerase (Bioline, London, UK). The primer sets used for expression analysis are listed in Table 1. Before RT-PCR, pilot experiments were performed to establish cycle number for the final analysis [27] and template dilutions to ensure that primer efficiency was as close as possible to 100% for all primer pairs. PCR conditions adopted were: 94°C 30 sec, 52°C 60 sec, 72°C 80 sec, 30 cycles and a final extension step of 10 min at 72°C.

The PCR products, after separation on an agarose gel, were acquired using a Kodak digital science 1D image analysis program (Eastman Kodak, Rochester, NY, USA). Densitometric analysis was performed using ImageJ software (http://imagej.nih.gov/ij). The expression level of all genes examined was normalized against the *ACT1* gene, which was used as an internal housekeeping control gene. All experiments were repeated in triplicate. Statistical analysis of the data was performed by analysis of variance (One-Way-ANOVA) using Bonferroni’s multiple comparison post-test. A *P-value* <0.05 was considered significant.

#### Replacement of the *C*. *africana* mutated *HXK1* gene by direct transformation with a wild-type *HXK1* fragment from *C*. *albicans*

The DNA region carrying the mutation in the *C*. *africana* CBS11016 *HXK1* gene was replaced using a 687 bp *HXK1*-fragment obtained from the *C*. *albicans* reference strain ATCC10231. The fragment was amplified in vitro using the following primer set: HXK1tr-fw GACTAGCATTAGTGGGTTGCG and HXK1-Rb CACCCAGCAGAATACACCG and the purified fragment was used to transform *C*. *africana* reference strain CBS11016 using the standard lithium acetate transformation method described previously [28] with the following modifications: transformants were recovered for 8h shaking in liquid YEPD media and then plated on SC media with 2% GlcNAc as the sole carbon source. Transformant colonies that were able to grow on the GlcNAc plates (after 5 days of growth) were selected and verified by sequencing. The MGC1 strain is a resulting verified transformant of the *C*. *africana* parent containing the wild-type *C*. *albicans HXK1* fragment.

## Results

### Phenotypic tests

With the exception of the *C*. *albicans* control strain ATCC10231, *C*. *africana* isolates UPV/EHU 97135 and CBS11016 did not grow in tubes in the presence of different concentrations of GlcNAc after 28 days of incubation at 30°C. However, all fungal isolates were able to grow in liquid medium containing both galactose and GlcNAc after 24h of incubation.

Morphologically, considerable differences were observed between *C*. *albicans* and *C*. *africana*. After 8h of incubation in different GlcNAc concentrations (ranging from 0.195–100 mM), *C*. *africana* strains showed mostly yeast-like forms and a small number of germ tubes and pseudohyphae (~30% of the cells) after 24h (Fig 1; showing representative images in 5 mM GlcNAc). We note that the Spanish isolate UPV/EHU 97135 showed yeast cells arranged in irregular grape-like clusters (Fig 1).

*C*. *albicans* ATCC10231 formed germ-tubes efficiently after 2.5h of incubation in GlcNAc and after 8h the culture consisted nearly exclusively of long and short pseudohyphae (Fig 1).

**Fig 2:**
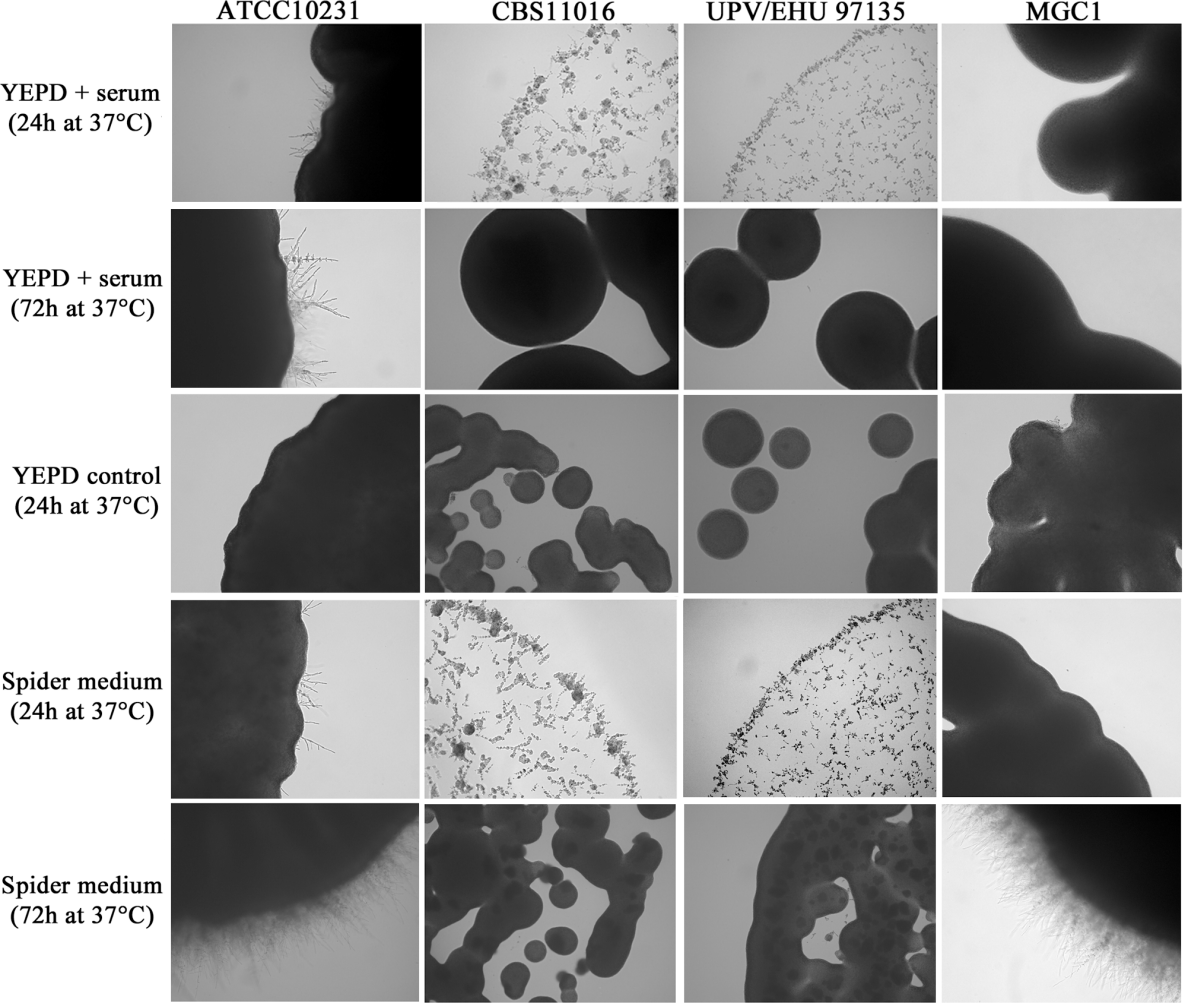
Microscopic characteristics of *C*. *albicans* and *C*. *africana* isolates grown on YEPD plus 10% serum and Spider medium at 37°C for 24 and 72h. YEPD control is also shown at 24h (at 72h the YEPD control was nearly identical to the 24h time point and thus is not shown). Images were taken from the first dilution (inoculum size ~10^4^ cells/μl). Magnification 20x.

The degree of filamentation was also analyzed in specific solid filament-inducing media and the data revealed that after 24h of incubation at 30°C and 37°C on Spider medium and YEPD plus 10% serum, the *C*. *albicans* reference strain began to form hyphae (Fig 2).

**Fig 3:**
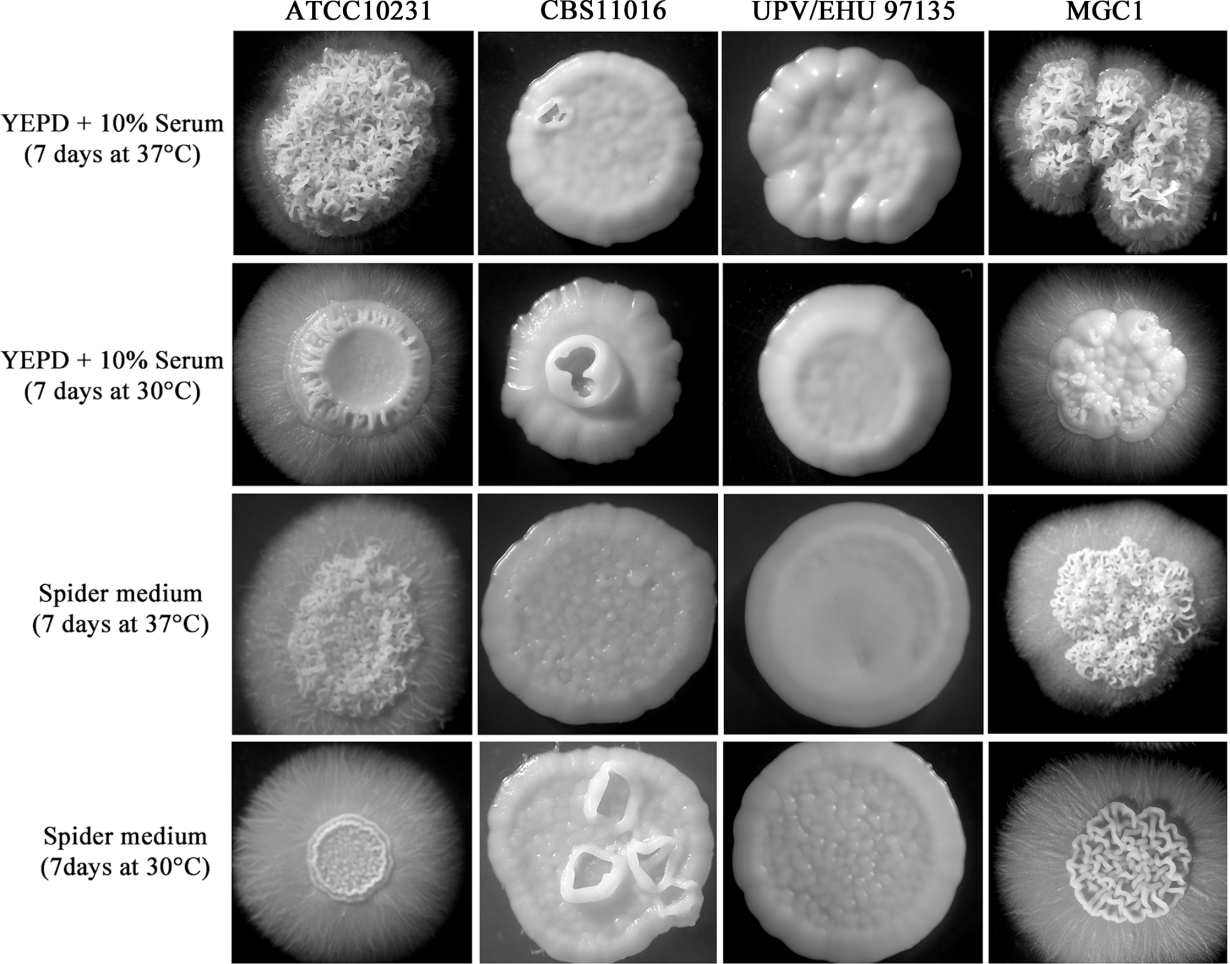
Morphologies of *C*. *albicans* and *C*. *africana* colonies obtained in this study after 7 days of incubation at 30°C and 37°C on YEPD plus 10% serum and Spider medium.

Upon microscopic examination, the colonies were composed mainly of pseudohyphal elements and after 7 days of incubation they were completely surrounded by hyphal outgrowth (Fig 3).

On the same agar media, *C*. *africana* isolates also showed increased filamentation after 24h at 30°C and 37°C, but their growth was delayed compared to *C*. *albicans* (Fig 2). The cultures were composed of hyphal forms (predominantly pseudohyphae) and budding yeast cells. On standard YEPD agar plates, used as a control, the colonies of *C*. *africana* strains were all smooth (Fig 2). After 48h of incubation on YEPD plus serum, *C*. *africana* isolates were mainly in the yeast form and their colonies appeared smooth, while in Spider medium, hyphae were abundant. After 72h at 30°C and 37°C, the colonies grown on YEPD plus serum were completely smooth, and in Spider medium, there was a notable increase of the yeast form. In contrast to *C*. *albicans*, after 7 days of incubation on these filament-inducing media, *C*. *africana* isolates showed only smooth colonies (Fig 3). Nevertheless, a high level of filamentation was observed in water plus 10% fetal bovine serum (vol/vol) at 30°C up to 7 days. In these conditions, *C*. *africana* produced filaments after 1h and no evident conversion back to the yeast form was observed during that time.

### Genetic and expression analyses

**Fig 4:**
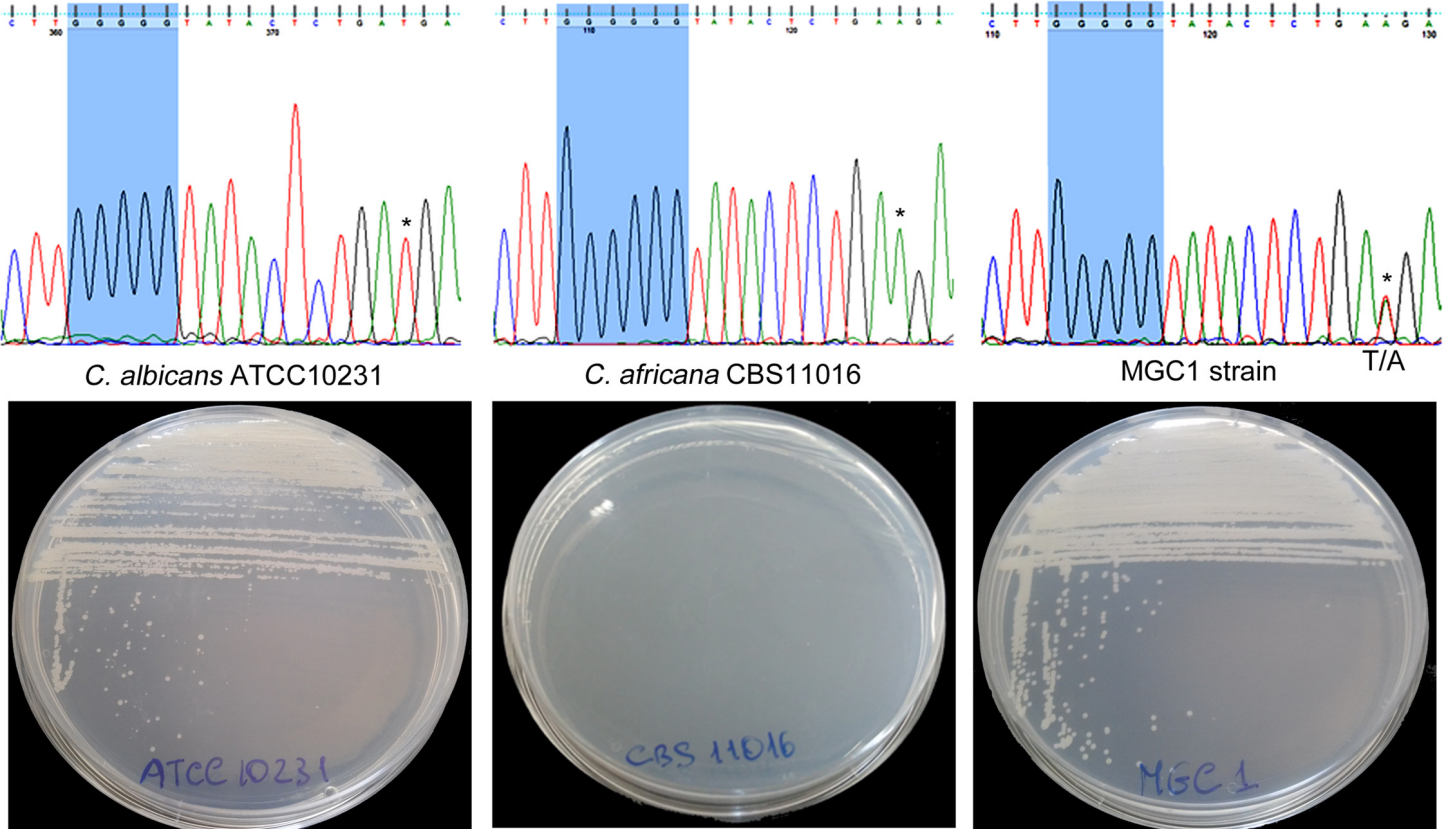
Partial *HXK1-*gene sequencing of *C*. *albicans* ATCC10231, *C*. *africana* CBS11016 and MGC1 strain showing the region containing the guanine insertion. In the MGC1 strain, a heterozygous site containing both *C*. *albicans* and *C*. *africana* specific nucleotides is also shown (* indicates the *C*. *albicans* and *C*. *africana* peaks that overlap in the heterozygous site). The respective growth of the strains in YNB medium plus GlcNAc is also shown.

DNA sequence analysis of *C*. *africana NGT1*, *HXK1*, *NAG1* and *DAC1* genes revealed a number of characteristic nucleotide substitutions including a unique and distinctive nucleotide (guanine) insertion (Fig 4) that generated a stop codon (TGA) 154 bp downstream of the ATG start codon of the *HXK1* gene. This mutation was also found in other *C*. *africana* strains from different geographical origins (ten Chinese strains and one German strain) [3, 21] included in this study. On the contrary, the *C*. *albicans* ATCC10231 strain lacked this polymorphism (Fig 4).

To verify whether the guanine (G) insertion was directly responsible for the inability to assimilate GlcNAc by *C*. *africana*, the DNA region containing this mutation was replaced in the CBS11016 *C*. *africana* strain using a specific 687 bp *HXK1*-fragment derived from the *C*. *albicans* reference strain. Partial sequencing of the *HXK1* gene of the resulting transformant (designated MGC1) confirmed the absence of the distinctive G insertion and the presence of both *C*. *albicans* and *C*. *africana* alleles as demonstrated by heterozygous sites (Fig 4).

Phenotypically, the MGC1 strain was able to grow in media containing GlcNAc as the sole carbon source (Fig 4) and its colonies resembled those of the *C*. *albicans* ATCC10231 strain when grown under filament-inducing conditions (Fig 3).

Comparison of DNA sequencing results of the *NGT1* gene and the orf19.5392 *C*. *albicans* reference sequence showed a complete coding sequence with a single non-synonymous substitution (*1010A→G*), which replaces lysine (K) with arginine (R) within the Ngt1 protein. This neutral substitution was the only mutation detected in the *NGT1* gene of our *C*. *africana* isolates.

Sequence alignment of the *DAC1* genes revealed several nucleotide substitutions, most of which were silent or neutral mutations in the respective proteins. Only one, the *640A→G* substitution, caused the replacement of an aspartic acid (D) with asparagine (N) within the *C*. *africana* Dac1 protein.

Regarding the *NAG1* gene, although several base substitutions were detected, two in particular, located at nt 334 and 443, caused the amino acidic substitution Glu→Lys and Ala→Thr at position 112 and 145, respectively, in all *C*. *africana* Nag1 proteins. Nevertheless, the putative 3D structure, predicted by comparative homology modeling (http://toolkit.tuebingen.mpg.de/hhpred) using the *Escherichia coli* glucosamine 6-phosphate deaminase protein (PDB: 1HOR) as a template, revealed no structural differences (data not shown).

**Fig 5:**
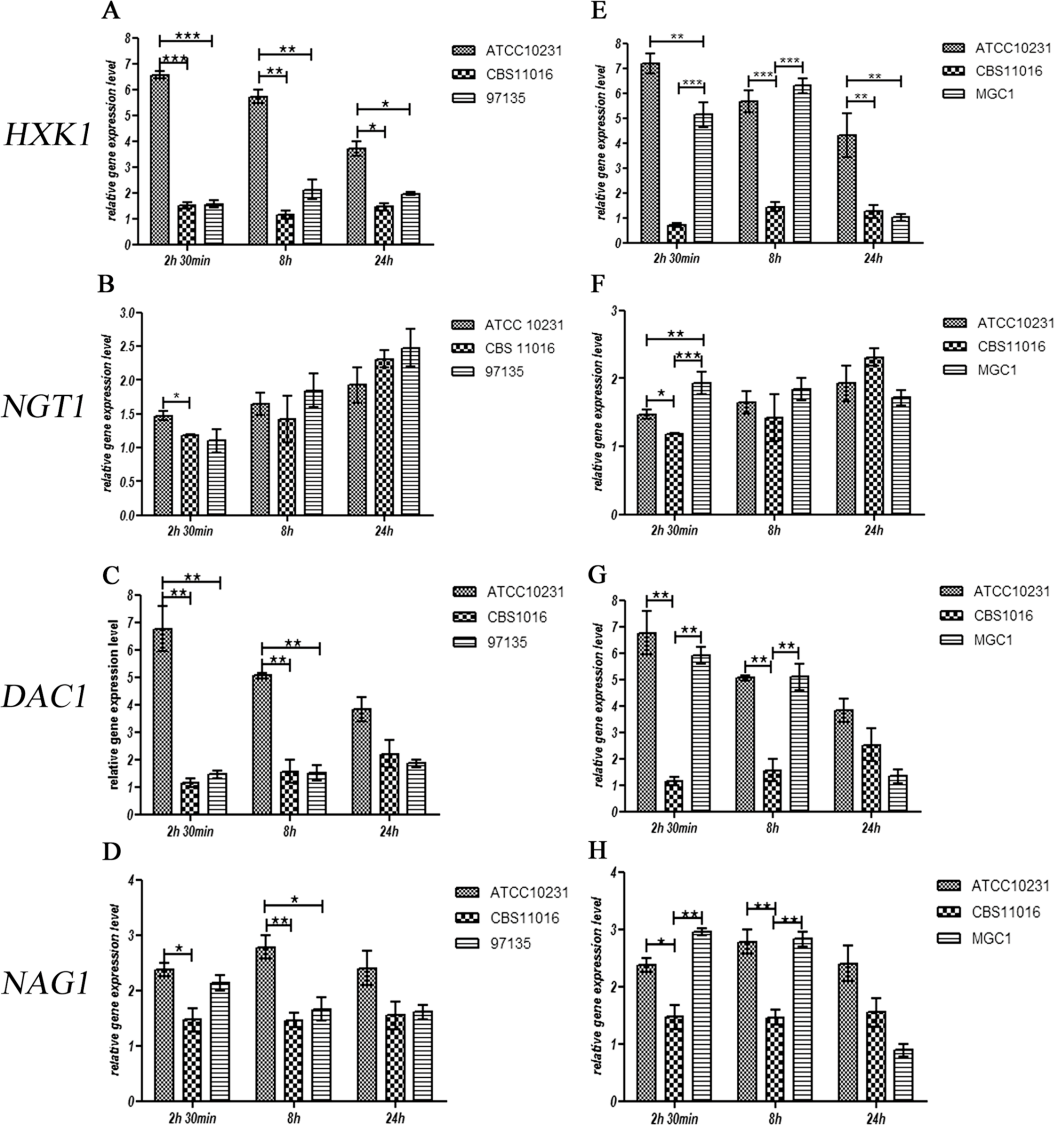
Effect of GlcNAc on mRNA expression levels of the *HXK1*, *NGT1*, *DAC1* and *NAG1* genes in *C*. *albicans* ATCC10231, *C*. *africana* CBS1016, *C*. *africana* UPV/EHU 97135 (A-D) and the MGC1 strain (E-H). Values are normalized to *ACT1* as an internal control. Data are expressed as fold changes with respect to untreated cells. The experiments were performed in triplicate and results are expressed as means ± SEM. Statistical analysis were performed using a One-Way-ANOVA with Bonferroni’s Multiple comparison post-test. A *P value* < 0.05 was considered significant.

The transcription of all genes involved in GlcNAc catabolism was also examined indicating that, in *C*. *albicans* ATCC10231, the presence of GlcNAc in the medium was able to evoke a strong initial induction of expression of the *HXK1* gene, which decreased progressively over time (Fig 5A).

Different results were obtained from *C*. *africana* strains in which the level of *HXK1* gene expression was much lower, compared with data obtained from *C*. *albicans* strain (P-value <0.05) (Fig 5A). These results suggest that the presence of a premature stop codon in the *C*. *africana HXK1* mRNA leads to an instable transcript that is rapidly degraded.

Previous studies showed that in *C*. *albicans HXK1* null mutants, the expression levels of the others genes involved in GlcNAc catabolism are altered [24, 29]. To better understand the effect of the naturally occurring *C*. *africana* non-functional *HXK1* gene, the expression levels of *NGT1*, *DAC1*, and *NAG1* genes were also investigated. The transcription of the GlcNAc transporter, in all *Candida* strains, does not seem to be impaired by the integrity of the *HXK1* gene. As shown in Fig 5B, the comparative analysis among *Candida* strains showed no significant differences between *NGT1* mRNA levels.

The effect on *DAC1* and *NAG1* transcriptional rate was significantly different; in fact, in *C*. *africana* strains, the transcriptional level of the *DAC1* gene was much lower at 2.5h and 8h compared to *C*. *albicans* (Fig 5C). The effect exerted on *NAG1* expression was less pronounced (although statistically significant), and very similar to that observed for the *DAC1* gene expression (Fig 5D).

In an attempt to clarify whether the differences observed at the transcriptional level for these selected genes were linked to a non-functional *HXK1* gene, the mutated allele in *C*. *africana* CBS11016 strain was replaced by homologous recombination using a functional *HXK1* gene derived from *C*. *albicans*. The resulting transformant (strain MGC1) displayed a full restoration of the *HXK1* gene expression that was very similar to that observed for the *C*. *albicans* wild-type strain (Fig 5E). However the *HXK1* expression levels decreased drastically at 24h, reaching a level similar to that observed for *C*. *africana* CBS11016 (Fig 5E). For the *NGT1* gene, results showed a possible independence of its transcription from the *HXK1* gene. In fact it is evident that the restoration of the functional *HXK1* allele in *C*. *africana* had no influence on the GlcNAC transporter *NGT1* in this timeframe (Fig 5F). On the contrary, the transcription of the *DAC1* and *NAG1* genes in *C*. *africana* appears to be considerably influenced by *HXK1* restoration; in fact, comparison of *DAC1* mRNA levels between the *C*. *africana* CBS11016 strain and the MGC1 strain highlights a strong effect exerted by a functional *HXK1*, producing a level of *DAC1* transcripts that is very similar to that of the *C*. *albicans* wild-type strain. Nevertheless, no differences were observed among the three *Candida* strains at 24h (Fig 5G).

Similar results were also obtained for *NAG1* gene expression (Fig 5H).

## Discussions

In last few years, many epidemiological studies reporting the clinical isolation of *C*. *africana* have appeared in the scientific literature and the data obtained revealed that this fungus is particularly adapted to colonize human genitalia and infects mainly such organs [9, 17, 19, 21, 30, 31, 32, 33, 34].

The propensity to cause genital infections may be the result of its unique and distinctive genetic background [7] which makes this fungus particularly interesting to study [1]. In fact, unlike typical *C*. *albicans* isolates, *C*. *africana* isolates display a reduced virulence, possess a limited pathogenic potential [17, 35] and display a number of phenotypic defects including the inability to metabolize several organic compounds [3]. Therefore, the existence of this natural mutant within the *C*. *albicans* population [10] offers a powerful model for understanding the molecular basis of pathogenicity, phenotypic diversification and adaptation to mammalian hosts for pathogenic members of the *Candida* clade.

Through this study, we were able to demonstrate that the lack of GlcNAc utilization by *C*. *africana* is due to a single nucleotide (G residue) insertion resulting in a frameshift mutation that, created a premature stop codon within the *HXK1*-gene encoding GlcNAc kinase. Thus *C*. *africana* can be considered a natural *HXK1* mutant as also evidenced by positive growth of our isolates in medium containing galactose plus GlcNAc, which impairs the development of the strains lacking the *DAC1* and/or *NAG1* genes, but not of the *HXK1* mutants [14, 24]. In addition, the replacement of the mutated *HXK1* allele using a functional allele from *C*. *albicans*, restored the gene function and GlcNAc assimilation in *C*. *africana* (Fig 4)

Previous studies have already genetically well-characterized the GlcNAc catabolic pathway in *C*. *albicans* [24, 29, 36] and revealed an important role of the NAG regulon in the biology and virulence of this fungus, especially regarding the function of the *HXK1* gene, which seems to be involved in various cellular processes in this species [29].

Disruption of the entire NAG cluster in *C*. *albicans* revealed some interesting phenotypic characteristics including the ability of the triple mutant to hyperfilament under stress-induced conditions [36]. This intrinsic morphological property has also been reported for *HXK1* null mutants [29, 37] and further confirmed in this study for *C*. *africana*.

Rao et al. [29] reported that the homozygous *HXK1* mutant, after 2 days of incubation at 30°C on YEPD plus serum, started to form wrinkled colonies which were completely surrounded by hyphae after 6 days in such conditions. Similar results were also obtained in a separate study [37] and are, in part, consistent with our data. In fact we observed that *C*. *africana* strains showed a strong increase in hyphal formation on solid media during the first 24h of incubation but their colonies soon turned smooth and no filamentation was observed after 7 days of incubation (Fig 3). However *C*. *africana* was able to filament abundantly in 10% serum in water; a similar behavior was also previously observed in *Candida dubliniensis*, a species phylogenetically closely related to *C*. *albicans* that fails to form hyphae in YEPD supplemented with 10% serum but not in nutrient-poor media [38]. Therefore, the strong tendency of *C*. *africana* to hyperfilament rapidly and quickly revert to the yeast-phase in specific rich agar media raises the hypothesis that nutrient-sensing mechanisms in this biovariant could be differentially regulated compared with *C*. *albicans* and may partly explain its low degree of virulence and pathogenicity observed in recent studies [17, 35].

In our study, differences were also seen in gene expression profiles. In fact although the expression levels of the *HXK1* gene were lower in all *C*. *africana* isolates (Fig 5A), considerable differences were observed regarding other genes involved in GlcNAc metabolism except *NGT1* whose expression, in agreement with previous studies, does not appear to be controlled by *HXK1* [24, 39]. Our data (Fig 5) highlight a strong influence of a functional GlcNAc kinase in the transcription of both *DAC1* and *NAG1* genes. These results are in accord with a conceptual scheme presented by Rao et al. [29] in which the *HXK1* gene plays a pivotal role in different cellular processes including *DAC1* and *NAG1* expression, and partially in accord with other published data [24] in which a *C*. *albicans HXK1* null mutant showed a reduced, but consistent transcriptional level for these genes, especially for the *NAG1* gene. Overall these data confirm that the Hxk1 enzyme could play additional roles other than its traditional metabolic role as a kinase [29].

We believe that the existence of a group of strains, referred as *C*. *africana*, within *C*. *albicans* populations, with distinct properties such as reduced virulence and pathogenicity, suggests the possibility for the presence of other novel biological functions, not only for the *HXK1* gene, but also for other aspects of the biology and genetics of *C*. *albicans*. Therefore *C*. *africana* could represent a useful natural model for future comparative genomics and transcriptomics studies in order to better understand the molecular mechanisms that make *C*. *albicans* the most predominant fungal pathogen for humans.
